# Arbovirus Transmission Predictions Are Affected by Both Temperature Data Source and Modeling Methodologies across Cities in Colombia

**DOI:** 10.3390/microorganisms11051249

**Published:** 2023-05-09

**Authors:** Víctor Hugo Peña-García, Jeffrey C. Luvall, Rebecca C. Christofferson

**Affiliations:** 1 Programa de Estudio y Control de Enfermedades Tropicales (PECET), Universidad de Antioquia, Medellín 50010, Colombia; 2 Marshall Space Flight Center, National Aeronautics Space Administration (NASA), Huntsville, AL 35824, USA; 3 Department of Pathobiological Sciences, School of Veterinary Medicine, Louisiana State University, Baton Rouge, LA 70803, USA

**Keywords:** arbovirus, chikungunya, dengue, aedes aegypti, basic reproduction number, temperature, micro-environment, Colombia

## Abstract

Weather variables has been described as major drivers of vector proliferation and arbovirus transmission. Among them, temperature has consistently been found to be impactful in transmission dynamics, and models that incorporate temperature have been widely used to evaluate and forecast transmission or arboviruses like dengue, zika, or chikungunya virus. Further, there is growing evidence of the importance of micro-environmental temperatures in driving transmission of *Aedes aegypti-*borne viruses, as these mosquitoes tend to live within domiciles. Yet there is still a considerable gap in our understanding of how accounting for micro-environmental temperatures in models varies from the use of other widely-used, macro-level temperature measures. This effort combines field-collected data of both indoor and outdoor household associated temperatures and weather station temperature data from three Colombian cities to describe the relationship between the measures representing temperature at the micro- and macro-levels. These data indicate that weather station data may not accurately capture the temperature profiles of indoor micro-environments. However, using these data sources, the basic reproductive number for arboviruses was calculated by means of three modeling efforts to investigate whether temperature measure differences translated to differential transmission predictions. Across all three cities, it was determined that the modeling method was more often impactful rather than the temperature data-source, though no consistent pattern was immediately clear. This suggests that temperature data sources and modeling methods are important for precision in arbovirus transmission predictions, and more studies are needed to parse out this complex interaction.

## 1. Introduction

Urban arboviruses like dengue (DENV), chikungunya (CHIKV), Zika (ZIKV), and yellow fever virus (YFV) continue to be a concern for the global health community, as they consistently are the cause of significant morbidity (and, in some cases, mortality), especially in tropical regions such as South America [1]. These viruses are all transmitted by *Aedes aegypti* and *Ae. albopictus. Ae. aegypti* has been identified as the primary vector in urban settings and the primary vector in most outbreaks [2]. Despite vector control efforts in affected regions, incidence of *Ae.* aegypti-borne arboviruses has continued to increase over recent decades [3,4,5,6]. Further, introductions of ZIKV and CHIKV into the western hemisphere, where they had not previously occurred, caused millions of cases and significant, long-term health issues for affected populations (reviewed in [7]). These arbovirus events continue to underpin the need for a more detailed understanding of transmission, including the combined and individual processes of both vector biology and arbovirus dynamics that drive transmission patterns.

It has been well-documented that the life traits of mosquito vectors are temperature-dependent [8]. For example, temperature governs mosquito egg survival and the rate of development among immature adult stages [8,9], body size [10,11] and mortality rates [12]. Also, many studies have demonstrated that some infection- and transmission-related variables of the mosquito-virus interactions are temperature-dependent, such as vector competence, the extrinsic incubation period (EIP), [13,14,15,16] and biting rate [17,18]. With these and other data, researchers have estimated the optimal ranges for mosquito development and DENV transmission are around 20–30 °C [10,19,20] and approximately 28 °C for ZIKV [21] and 29 °C for CHIKV [22].

With the increasing understanding of the importance and impact of temperature-dependence in arbovirus transmission, it is not surprising that a multitude of temperature-sensitive models have been developed to predict and estimate the burden of arbovirus transmission. Most of these efforts focus on estimating the basic reproduction number (*R*_0_), which provides a measure of transmission success in a naive population. Several models have adapted the derivation of *R*_0_ to explicitly include the biological traits of mosquito populations, and this was originally introduced through a concept known as vectorial capacity [23,24]. 

Many studies estimating mosquito life traits or transmission-related parameters use broad measures of temperature like those from meteorological weather stations or their derived interpolations [25,26]. However, in addition to demonstrating the importance of accounting for temperature, studies have recently begun to highlight the importance of considering aspects of the micro-climate and micro-environment in mosquito abundance [27], distribution [28], and arbovirus transmission [29,30,31]. How well broad estimates such as weather station data reflect temperature-dependent dynamics at the resolution of the microenvironment (such as indoors, where *Ae. aegypti* reside) is something that is poorly understood. Studies have demonstrated that transmission of DENV occurs primarily within and among households, due in part to the anthropophilic behavior of *Ae. aegypti* [32]. Thus, it is likely this household-centric model would hold for other *Ae. aegypti*-borne viruses like CHIKV and ZIKV. This further confirms that the micro-environment should be considered when investigating drivers of transmission.

Colombia is endemic for DENV, and now for ZIKV and CHIKV. Colombia currently reports thousands of weekly cases due to its weather and socioeconomic conditions, which are suitable for transmission year-round [25,33,34]. Herein, field collected data at the household level of both indoor and outdoor temperatures as well as weather station data from Colombian cities are combined with investigations of model methods of varying complexity, to describe the impact of including different measures of temperature on estimates of arbovirus transmission. 

## 2. Methods

### 2.1. Study Sites and Household Associated Temperature Monitoring

**Figure 1:**
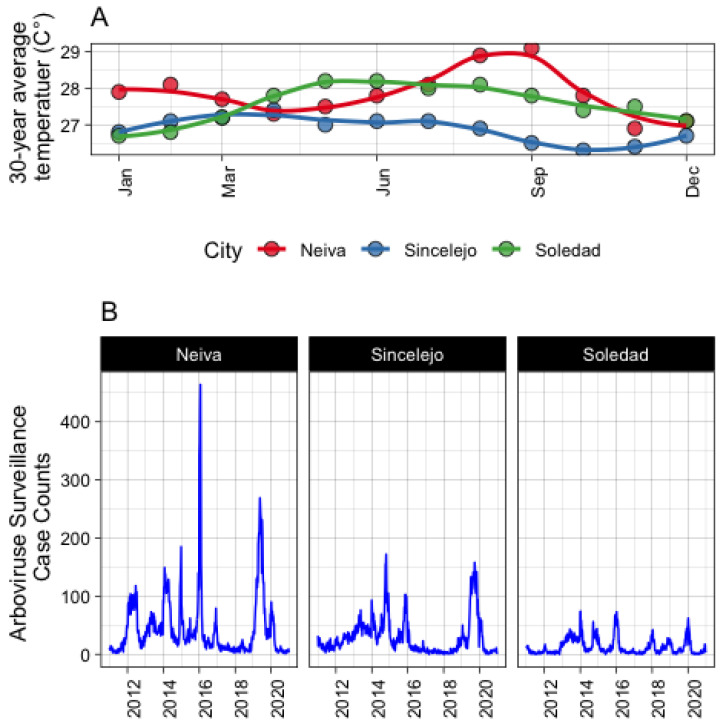
(**A**) Monthly 30-year average temperature according to data reported by IDEAM (Instituto de hidrología, meteorología y estudios ambientales, in Spanish), the governmental institute which monitors and records country-wide environmental variables. (**B**) Total arbovirus reported cases by SIVIGILA during the last decade.

This study was performed in three cities from Colombia with IRB ethics approval from Louisiana State University. The cities were selected based on historical differences in both temperature and available epidemiological data for arbovirus transmission patterns (Figure 1), despite having similar mean temperature patterns according to broader weather station data. Patterns of arbovirus transmission were observed from *Sistema Nacional de Vigilancia en Salud Pública* (SIVIGILA) data, the national public health surveillance system in Colombia that publishes reported cases of arbovirus infections from territorial entities. Each city represents a different ecological setting: Neiva (2.9345° N, 75.2809° W) is a land-locked city with an elevation of 442 m above sea level (masl) located in the middle of two mountain ranges (central and eastern) where the river Magdalena circulates. Sincelejo (9.3046° N, 75.3906° W) is located in north-east Colombia in the Colombian Caribbean region approximately 30 km from the sea with an elevation of 213 masl. Soledad (10.9215° N, 74.7688° W) constitutes a single urban area with Barranquilla and is next to the sea. Given that housing conditions are not particularly different between cities (personal observation), these sites were chosen in order to determine whether weather station data accurately represented household-level temperature and to what extent differences in household associated temperature (if any) contributed to differences in estimated arbovirus transmission. 

**Figure 2:**
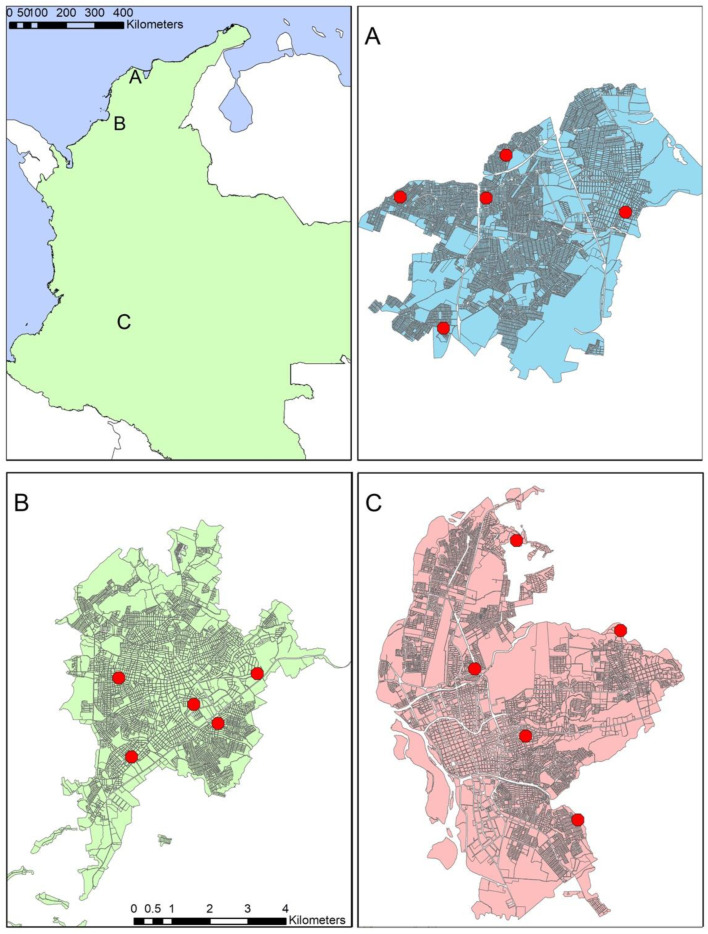
Location of the three cities in Colombia (top left panel): Soledad (**A**), Neiva (**B**), and Sincelejo (**C**). Red dots indicate approximate location of temperature data logger installation (approximate geographic resolution for purposes of maintaining anonymity).

Household-associated measures of temperature were measured both indoors and outdoors for the three cities in Colombia (details below) to represent micro-environmental temperatures. Weather station data was also collected to represent “broader” measures of temperature. Weather stations were located in or near each city. Each of these data sources was used to estimate arbovirus transmission with three published models (details below). Transmission estimates based on data source—indoor-household temperatures (IHT), outdoor-household temperatures (OHT), or weather station temperatures (WST)—or modeling methodology. Temperature data loggers (HOBO^®^ MX2301) were installed in five households in each of the three cities (Figure 2). Inside each house, the data logger was positioned in a place where inhabitants self-reported the presence of mosquitoes, usually reported as the bedroom or living room. Outdoor data loggers were installed in a backyard when available or the outer side of a wall (such as stoop or porch) where they were readily accessible and relatively secure. Once installed, the data loggers were programmed to record temperature every four hours at 00:00, 04:00, 08:00, 12:00, 16:00 and 20:00 h each day. The loggers were installed between late July and early August of 2019 for the three cities and left to record until their removal in June—August of 2020. Data were exported as Microsoft Excel files (Microsoft Corp., Redmond, WA, USA, version 16.72) by using an iPad device (Apple Inc., Cupertino, CA, USA) with the HOBOmobile application (ONSET, Cape Cod, MA, USA). Due to the national shutdown during the early stages of the pandemic, it was not possible to troubleshoot loggers and thus the data for some devices are incomplete (Supporting Figure S1.1, Table S1.1). 

### 2.2. Community Level Weather Stations Temperature (WST) Data

For comparisons, temperature readings recorded by meteorological weather stations located inside the city or the closest reporting station to that city were obtained. For example, while data was available from within Neiva, data for Sincelejo was approximated from a weather station ~12 km East (Corozal). This data was downloaded from the webpage of governmental entity IDEAM (Instituto de hidrología, meteorología y estudios ambientales, in Spanish, webpage http://www.ideam.gov.co/, accessed on 4 January 2021). Unfortunately, data from Soledad or nearby neighboring cities were unavailable for the time period of this study.

### 2.3. Estimation of Temperature-Dependent Arbovirus Transmission Dynamics 

Temperature data was used to estimate the potential for an outbreak of *Aedes-*borne arboviruses using three published methods that were developed to account for the impact of temperature on transmission dynamics and which have varying elements of complexity. The basic reproductive number (*R*_0_) is a measure of the potential for an infectious disease to persist in a complete susceptible population. While we recognize that a completely susceptible population is unlikely in an endemic region such as Colombia, we use the symbology used from the original methodologies published. 

The first method by Liu-Helmersson et al. was published in 2014 and was developed to estimate the relative vectorial capacity of dengue based on temperature [35]. They defined relative vectorial capacity (*rVC*) as vectorial capacity divided by the vector-to-human population ratio, where vectorial capacity was based on the classical equation developed by Ross-McDonald [23]. The equation for *R*_0_ derived from the *rVC* by Liu-Helmersson et al. is the least complex and based on relationships with temperature developed by other authors. The equation is at follows:

$$R_{0}=\frac{rVC}{T_{h}m}=\frac{a^{2}b_{h}b_{m}e^{-\mu_{m}n}}{\mu_{m}}*\frac{1}{T_{h}m}$$

where *rVC* is the relative vectorial capacity with the following parameters: a is the average daily vector biting rate, $b_{h}$ is the probability of vector to human transmission per bite, $b_{m}$ is the probability of human to vector infection per bite, n is the duration of the extrinsic incubation period, and $\mu_{m}$ is the vector mortality rate. Further, *T_h_* is the human infectious period, and *m* is the human-to-vector population ratio.

The second methodology is the mechanistic model developed by Mordecai et al. in 2017 [36] and has been used by others to estimate *Aedes*-borne transmission [21,37]. This methodology also uses a temperature-dependent modification of vectorial capacity to derive *R*_0_ with further complexities of the mosquito life cycle included:

R0=a2bce−μ/PDREFDpEAMDRNrμ312.

For this equation, a is the mosquito biting rate, b is the proportion of infectious bites infecting susceptible humans, c is the proportion of bites on infected humans that infects susceptible mosquitoes, μ is the adult mosquito mortality rate, PDR is taken as the inverse of the extrinsic incubation period, EFD is the number of eggs produced per female mosquito per day, $p_{EA}$ is the mosquito egg-to-adult survival probability, MDR is the immature mosquito development rate, N is the density of humans, and r is the human recovery rate (1/infectious period). The *R*_0_ values are rescaled with the value of 1 occurring at the unimodal peak [36].

Finally, a third approach published by Caminade et al. [38] based on the fundamentals of Turner et al. [39] was investigated. This approach involves a different set of equations than those Ross-MacDonald previously described and has the added complexity of two vector species (*Ae. aegypti* and *Ae. albopictus*) accounting for differences due to biological properties, as follows:

$$R_{0}=\sqrt{\hat{R_{11}}+\hat{R_{22}}}$$

where $\hat{R_{11}}=\left(\frac{b_{1}\beta_{1}a_{1}^{2}}{\mu_{1}}\right)\left(\frac{v_{1}}{v_{1}+\mu_{1}}\right)\left(\frac{\varphi_{1}^{2}m_{1}}{r}\right)$ and $\hat{R_{22}}=\left(\frac{b_{2}\beta_{2}a_{2}^{2}}{\mu_{2}}\right)\left(\frac{v_{2}}{v_{2}+\mu_{2}}\right)\left(\frac{\varphi_{2}^{2}m_{2}}{r}\right)R_{ij}$ is the average number of infectious vectors of type i produced by an infectious vector of type j, where 1 is related to *Ae. aegypti* and 2 is related to *Ae. albopictus*. The expression includes a as biting rates, μ as mortality rates, and 1/v as extrinsic incubation period, which rely on temperature data. The vector preferences (φ), transmission probabilities (b for vector to host and β for host to vector), and recovery rate (r) are assumed to be constant while vector to host ratios were estimated from a worldwide probability of occurrence reported by Kraemer and co-workers [2]. This method also includes a rescaling of *R*_0_ values based on mosquito density due to global geographic heterogeneities; however, we employed here the method of rescaling from [36] for comparisons as the geography considered herein is relatively contained.

Where possible, the same values for fixed parameters were used. For example, the human infectious period was defined as 7 days [38] and the same formulation for temperature dependent biting rate was used for Caminade and Lui- Helmersson methods. 

## 3. Data Analysis

Average temperatures were checked for normality to satisfy the assumptions of subsequent analyses. Data were confirmed to follow a normal distribution using the Kolmogorov-Smirnov test results to assess goodness of fit to normal distribution for the distribution of daily mean temperatures with the exception of Outdoor-Soledad (Figure S1.1). The WST was available only as a single measure per day (7 measures per week) and HOBO data also had missing data. To standardize the analyses across temperature data sources, the mean and standard deviations of daily temperatures from the three temperature sources (IHT, OHT, and WST) were calculated on a per-city basis for weekly temperature profiles. These profiles were then used to determine temperature-dependent parameter values and ultimately *R*_0_ estimates. 

The distribution of the values of common parameters across the three models were compared with respect to data source and model (where appropriate) using the *Kolmogorov-Smirnov test.* The proportion of weeks within each city where values of *R*_0_ higher than 1 were derived and compared with respect to data source and methodology. The “real-world” number of weeks with *R*_0_ > 1 was derived from historical data of total arbovirus cases reported in the SIVIGILA data (Figure 1). If the case count from week i to week (i + 1) was greater than 0, *R*_0i_ was coded as 1, otherwise it was coded as 0. The difference in the proportion of weeks predicted from each method/city combination and those estimated from SIVIGILA data were compared using a Chi-Square test for proportions. For some weeks, *R*_0_ > 1 was used rather than case counts for the following reasons: (1) case counts for arbovirus cases are generally lower than actual transmission given the high subclinical presentation numbers [40], and (2) the values herein were formulated from models designed to estimate *R*_0_, which assumes a completely susceptible population. As stated, Colombia is endemic for *Ae. aegypti-*borne viruses and there was concern that case count estimates would not represent this endemicity. Significance was assessed at the alpha = 0.05 level and all analyses were performed using R and R Studio (versions 4.2.1 and 2022.07.0 + 548, respectively).

### Visualization of Micro-Environmental Temperature on DENV Transmission

Land Surface Temperature (LST) maps were obtained for the three selected cities using Landsat-8 satellite imagery, as another indication of publicly available outside temperatures. At first, each of the complete Landsat-8 images were trimmed to the size and shape of the entire urban area of the given city. Those images with high presence of clouds were discarded and those with little cloud contamination (up to about 40% of the area) were processed by using the Landsat Quality Assessment ArcGIS Tools provided by USGS, United States [41,42]. Landsat-8-derived LST images were obtained by using the algorithm proposed by Avdan & Jovanovska (2016), which uses the Landsat-8 thermal infrared sensor Band 10 data and the NDVI, estimated with bands 4 and 5 from the same satellite [43]. LST images were assumed to be a macro-environmental representation of outdoor temperatures for each city. The relationship between indoor and outdoor temperatures was established by determining the association between the IHT from HOBO devices and the OHT from HOBO devices per city per month. Linear regressions defined these relationships with the mean daily-averaged OHT as the independent variable and the HOBO-measured daily-averaged IHT as the dependent variable. These relationships were then used to transform LST images (representing outdoor temperatures) into estimated indoor temperature maps in order to visualize the differences in accounting for micro-environment versus not across the geography of our study site. Temperature maps were then used to obtain *R*_0_ maps via each of the three methodologies previously described with their respective transformations when applied. The full range of values from the raster images was divided into four discrete categories representing quartile-defined ranges, to visualize areas with higher or minimum values. Additional statistics were estimated from each of the maps, including minimum and maximum values, mean and standard deviation, and the proportion of area containing values falling in each quartile.

## 4. Results

### 4.1. Differences among Temperature Data Sources and Resulting Parameters in Colombian Cities

**Figure 3:**
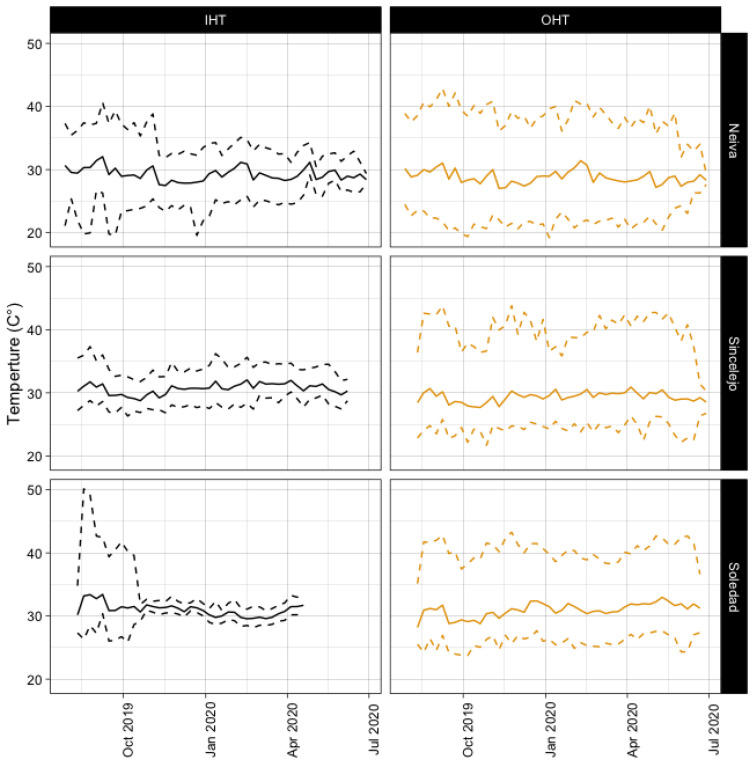
The mean (solid line) and min and max (dotted lines) for each city and household associated temperature, IHT, and OHT.

Temperature profiles differed among cities across data sources, with the one exception of IHT between Soledad and Sincelejo. When the source of data was considered, indoor temperatures displayed overall smaller ranges in temperature throughout the day (Figure 3 and Figure S1.1). This indicates that temperatures within the households were more consistent than outdoors, though the average temperatures were relatively similar (Supplemental Table S1.2).

**Figure 4:**
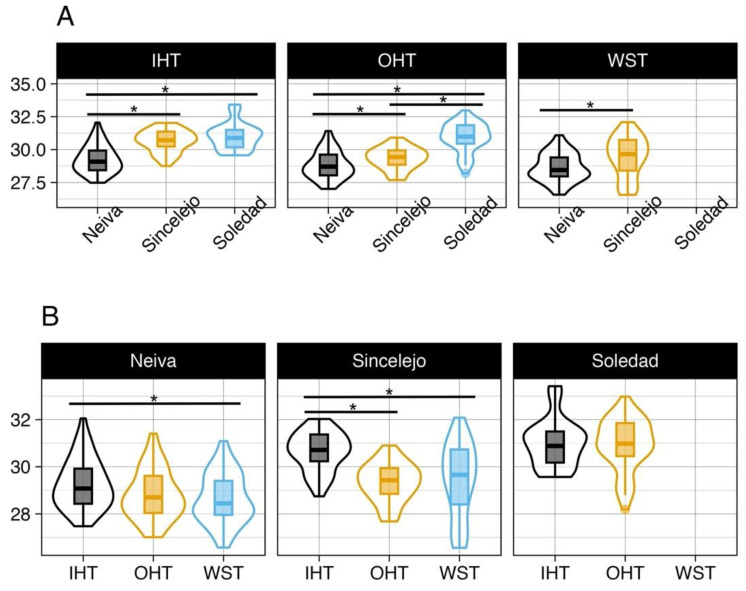
Distribution of the daily mean temperatures recorded by three methods for each city. (**A**) Comparisons demonstrate that the three cities had different temperature profiles more often than not. (**B**) Comparisons indicate that differences were more likely to exist between IHT, OHT, and WST data sources. * indicates significance at the *α* = 0.05 level.

Comparisons of data sources among the three cities demonstrated that the IHT distribution of Neiva was significantly different (and lower) than both Sincelejo and Soledad. OHTs were significantly different among all cities, while the WST was different between Neiva and Sincelejo (WSTs were unavailable for Soledad, Figure 4A). Unlike when individual households were compared, at the city level, there was no consistent pattern in temperature differences. For example, the distribution of daily mean IHTs were significantly different from the WST mean temperature for both Neiva and Sincelejo (WSTs were unavailable for Soledad). The OHT was less consistent as it was different from the WST for Neiva but not Sincelejo. The distribution of mean OHTs were different from the IHT for Sincelejo only. The IHT and OHT distributions were not different from one another in Soledad (Figure 4B). 

Next, the effects of temperature on life traits common to all three modeling methods across cities were compared. The parameters were biting rate (a), EIP (1/EIR), the probability of successful transmission from human to mosquito (proxy of vector competence, bm), and mosquito mortality (mu). Comparisons of the distributions of the individual parameters with respect to temperature data source within each city revealed that most often significant differences were attributable to the method used to estimate values rather than the data source (Supplemental Figures S2.1–S2.4). Notably, however, the WST did yield significantly different distributions of biting rate and EIP compared to the IHT in Neiva, across all three methods. Vector competence and mosquito mortality were only different with respect to the Caminade and Mordecai methods, however. Similarly in Sincelejo, the WST yielded significantly different distributions of biting rate, EIP, and mosquito mortality compared to the IHT across all three methods. Further, differences were noted in the distributions of biting rate, EIP, mosquito mortality, and mosquito density between the IHT and OHT for both Caminade and Mordecai methods, while the Liu-Helmersson method only resulted in differences in mosquito mortality and density between these two data sources in Sincelejo. No differences in parameter distributions were attributed to temperature data source for Soledad (Supplemental Figures S2.1–S2.4). 

**Figure 5:**
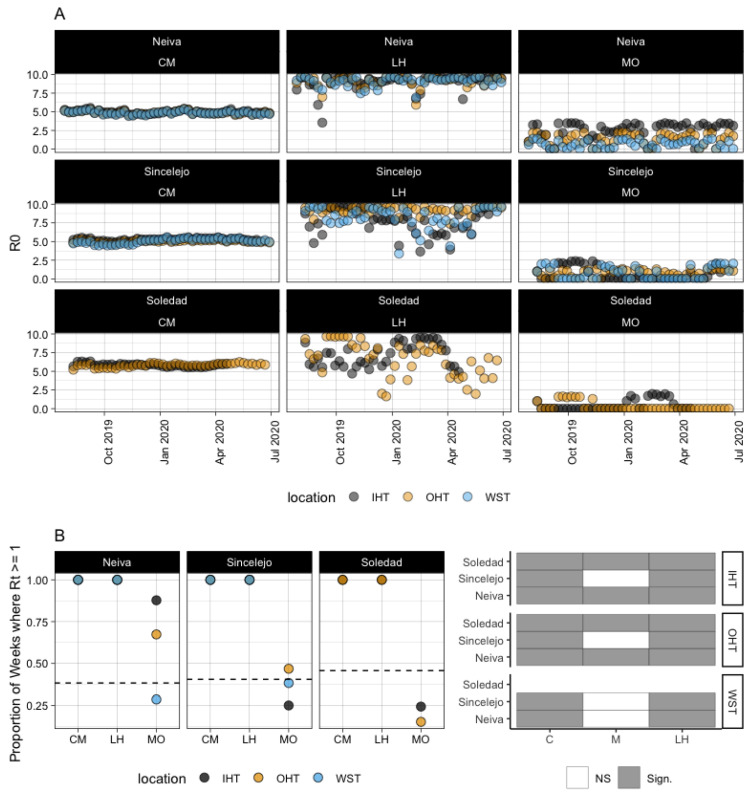
(**A**) Predicted *R*_0_ values by modeling method, city, and temperature data source. (**B**) The proportion of weeks where *R*_0_ > 1 for each city, temperature data source, and modeling method. Dotted lines represent the proportion of weeks where *R*_0_ > 1, derived from historical data of reported cases from SIVIGILA (Sistema Nacional de Vigilancia en Salud Pública in Spanish) for each respective city. The shaded grid on the far right indicates which comparisons of proportions of weeks where *R*_0_ > 1 were significantly different from historical estimates.

*R*_0_ was calculated using all three methods for each city over time using the three data sources (Figure 5A, Supplemental Figure S2.5). The Caminade method demonstrated little variability over the weeks, with *R*_0_ remaining at or above 5 for all three data sources and all three cities. The Liu-Helmersson and Mordecai methods have more variability; Liu-Helmersson tended to produce higher values. The Mordecai method was the only method to produce values of *R*_0_ less than 1. When compared across city, method, and data source, the distributions of *R*_0_ were significantly different except in the case of Soledad vs. Sincelejo (Mordecai method), Nevia vs. Sincelejo (Liu-Helmersson method) and Soledad vs. Sincelejo (Liu-Helmersson method) (Supplemental Figure S2.6). 

Case numbers from SIVIGILA were used to classify whether weeks had an increase in case counts relative to the week before. This proxy was used to determine the proportion of weeks where *R*_0_ could reasonably be assumed to be greater than or equal to 1 in historical data. It is important to note that *R*_0_ assumes a completely susceptible population, while Colombia is endemic. Using this proxy, we compared the number of weeks over the study period for each city that had positive case growth to the proportion of weeks from each method-data source-city combination with *R*_0_ greater than 1. From historical data, 38%, 40.5%, and 45.7% of weeks had positive case growth for Neiva, Sincelejo, and Soledad, respectively. Figure 5B demonstrates that all three methods estimated a much higher proportion of weeks across the board, with 100% of weeks having *R*_0_ > 1 for all combinations of cities and data sources for both the Caminade and Liu-Helmersson methods. However, the Mordecai method tended to have more variability, with 87.8% for Neiva-IHT, 67.3% for Neiva-OHT, 28.6% for Neiva-WST, 25.0% for Sincelejo-IHT, 46.8% for Sincelejo-OHT, 38.3% for Sincelejo-WST, 24.3% for Soledad-IHT, and 15.2% for Soledad-OHT. In fact, in half the comparisons of proportions, the estimates from Mordecai were not significantly different from the historical data (Figure 5B).

### 4.2. Visualization and Implications for Spatial Variability in R_0_ Estimates

**Figure 6:**
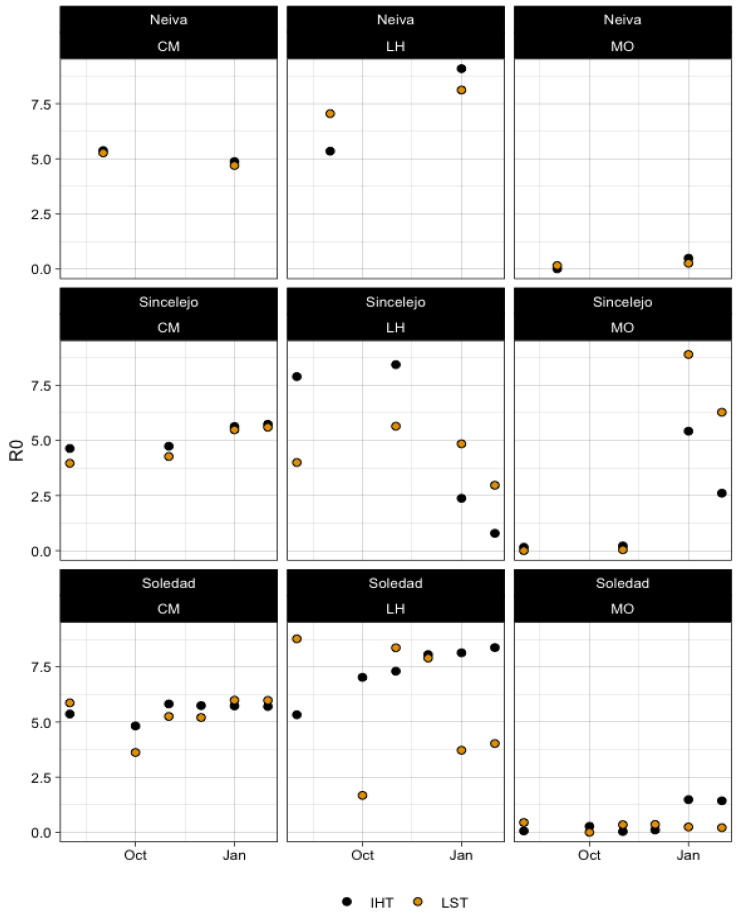
Average city-wide *R*_0_ values produced from LST compared to derived indoor temperature profiles across all three cities and methods.

An estimated indoor temperature map was derived from LST maps, where the IHT was directly estimated via linear models made from the relationship between the IHT and LST. The intercept and slope for each month as well as the respective determination coefficient are provided in Supplemental Table S1.2 and Figure S1.2. Average, city-wide *R*_0_ values were obtained for each method-data source combination for a common time period where satellite data was available (Figure 6). Similar to the IHT vs. OHT and WST data for the Caminade method, there was little difference in the IHT vs. LST estimates of *R*_0_, whereby each city had uniformly *R*_0_ values greater than 1. Overall, when assessed at a city-wide level, there was no consistent difference immediately observable between the two temperature measures of IHT vs. LST (Figure 6).

**Figure 7:**
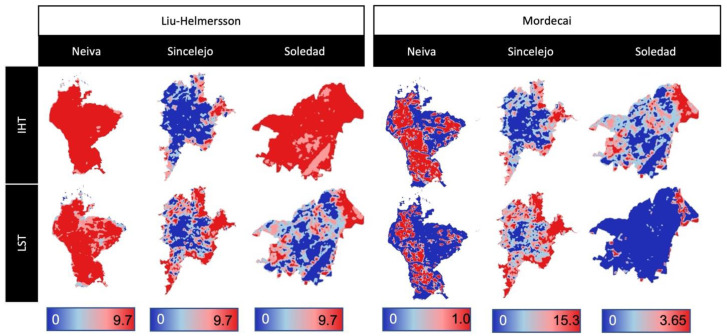
Maps of *R*_0_ values based on LST data for all three cities from January 2020, using the Liu-Helmersson and Mordecai methods for calculations.

Spatial analyses are sometimes used to determine the presence of risk hot-spots based on characteristics like temperature or ecology. Spatial *R*_0_ values were mapped for a common time point across all three cities where LSAT data were available (January 2020). Caminade maps produced little spatial variability and *R*_0_ values were universally over 1. However, there was some variability among the Liu-Helmersson and Mordecai *R*_0_ based on the LST data, though with variable magnitude (Figure 7). 

## 5. Discussion

The influence that temperature exerts on arbovirus transmission is well established and described in the scientific literature (reviewed in [44,45,46,47]). The fact that temperature affects most of the mosquito life traits as well as infection-related processes means that it is critical to account for this extrinsic factor when estimating virus transmission dynamics. There is a myriad of forecasting models that use temperature to inform predictions of transmission, risk, and/or burden of arboviruses and associated diseases. However, the fact that questions remain on how and to what extent temperature should be considered is apparent in the variability of complexity among models, as well as the number of parameters or other sources of variation on which to apply temperature dependence.

Given the anthropophilic behavior of *Ae. aegypti*, the amount of time a person usually spends in the house leads to the hypothesis that household associated transmission was an important contributor to total transmission. Indeed, several studies have confirmed indoor mosquito infection rates capable of contributing to arbovirus transmission [32,48,49,50,51,52] and studies have supported the idea that households are the foci of transmission in *Ae. aegypti*-arbovirus systems [53,54,55]. Thus, it is intuitive that household associated microenvironments are important to consider when predicting the dynamics of arbovirus transmission.

These data did indicate that the mean temperature of household associated data tended to be different from weather station associated data, and in most cases, the IHT was different from the OHT. The data also demonstrated that indoor temperatures were less variable compared to outdoor temperatures, even when the mean temperatures were similar. The differing ecologies of the cities may account for some of the variability. Soledad is a coastal city whereas Sincelejo is more land-locked, and Neiva is at a higher elevation. The interaction of these ecological differences and temperature remains to be studied in terms of effects on transmission, especially the effects of humidity [56]. There were observed effects on temperature across the three cities, but the results herein suggest that the modeling method had a larger impact on *R*_0_ estimates than differences in city. This indicates that micro-environmental temperatures—such as indoor household temperatures—may not be accurately assessed by the simple, convenient sampling of more distant measures like weather stations or satellite data. However, whether there is a cost-benefit of capturing micro-environmental temperatures remains murky.

*R*_0_ estimates calculated from the OHT and WST were not often different from one another, while the IHT did seem to differ from the other two data sources more often. On the surface, these results seem to suggest that micro-environmental, household associated temperature measures may not offer any additional insights for transmission predictions nor with comparisons with historical trends. Considering that *Ae. aegypti* are known to frequently live within domiciles, these results were surprising. However, the possible reasons for this are discussed here. First, it may in fact be that indoor temperatures do not represent the ambient temperature experienced by mosquitoes even within the house. Shaded areas or resting under furniture might alter the temperature experience of a mosquito compared to general ambient room temperature as measured by the HOBO devices. Second, this study and many modeling efforts often do not consider temperature fluctuations which have been found to effect within-vector virus dynamics, though the magnitude of these effects are variable [57,58,59]. Third, our study concentrates on the contribution of micro-environmental temperature differences only and does not consider other factors that contribute to arbovirus transmission such as human behaviors and vector control efforts [60,61]. Fourth, underreporting of arbovirus cases has been documented in Colombia [62,63,64], so the real extent of arbovirus burden may not be completely represented by SIVIGILA data [40]. Without field studies for direct case detection coincident with ecological studies, it is impossible to identify the full swath of variables that define transmission in specific contexts. Finally, this study was during the pandemic lockdown in Colombia and epidemiological behavior might not be indicative of “normal” [65,66] and declining cases were reported in some places [67,68] as well as a COVID-related potential for misdiagnosis [69,70]. The SARS-CoV-2 pandemic arrested much activity in Colombia in the middle of sampling time. Especially in Soledad, data were incomplete owing to device malfunction or other unidentified issues, which were exacerbated by an inability to return to sites as planned due to travel restrictions within Colombia. Inhabitants kindly removed the equipment at the end of the study time and returned to Medellín. Additionally, complete data were not available for the time of this study from weather stations from Soledad or nearby stations, which prevented us from comparing weather station data for that city. The same was true for LST data, which had its own restrictions due to cloud cover, etc. But it is important to note that newer and more sophisticated satellite products are becoming more available, which may encourage their use. Preliminarily, it does appear that if models can demonstrate variability in *R*_0_ predictions, the use of spatial data such as LST could be used to identify potential hotspots for arbovirus transmission and/or emergence. The pros and cons of each data source—none of which yielded complete data—should be weighed when assessing the avenues of investigation of this and similarly complex systems.

Climate change has become a major concern as increased temperatures may lead to expanded vector ranges, (re)emergence of arboviruses, and/or changes in established transmission patterns. Models have become an important toolset to gauge not only the potentially disastrous consequences of increased temperatures, but also to evaluate mitigation efforts. Despite the caveats of this current study, our study combines field and theoretical methods to demonstrate that temperatures from indoor microclimates vs. broader measures produce differential transmission patterns (compared to the IHT) across several mathematical prediction methods. This is an indication that indoor microenvironmental temperatures have the potential to drive transmission intensity. Precision in parameter inclusion and resolution needs to be prioritized to increase model acuity. However, these results do suggest that accounting for temperature-dependence in a more nuanced model parameterization might be necessary to capture temperature-dependence in transmission.

Of course, these data do not represent the totality of the micro-ecology of the *Ae. aegypti-*transmitted virus systems and more work is necessary to continue to interrogate the factors within the micro-environment that are critical for prediction precision. Further studies in these and other endemic cities are necessary to dissect the relationship between transmission and the micro-environment. This includes assessments of true arbovirus burden in the human population, and detailed ecological studies of the mosquito micro-habitat and factors associated with such. The true impact of temperature and how to best account for it in models continues to be an avenue of investigation.

## Data Availability

Relevant Data is available within the publication, referenced links, or supplemental data file.

## Supplementary Materials

The following supporting information can be downloaded at: https://www.mdpi.com/article/10.3390/microorganisms11051249/s1. File S1: Figure S1.1: Values recorded by temperature data loggers in the three cities at six different hours along the day; Figure S1.2: Regression analyses performed on HOBO-recorded outdoor and indoor daily averages for each month and each city; Table S1.1: Results of *t*-test comparison analyses between daily mean temperature values taken indoor (IHT), outdoor (OHT) and weather stations (WST). *p*-values in bold italic are statistically significant at 95% confidence; Table S1.2: Summary of linear associations between OHT and IHT daily-averaged temperatures. (Model: IHT = OHT). File S2: Figure S2.1: Temperature-dependent estimate of biting rate of *Ae. aegypti*; Figure S2.2: Temperature-dependent estimate of the probability of successful transmission from human to mosquito (of *Ae. aegypti*) as an estimate for vector competence; Figure S2.3: Temperature-dependent estimate of the extrinsic incubation period of *Ae. aegypti*; Figure S2.4: Temperature-dependent estimate of the mosquito mortality of *Ae. aegypti*; Figure S2.5: Temperature-dependent estimate of the *R_0_* for each method; Figure S2.6: Distributions of the *R_0_* values per city x data source x modeling method. All distributions were significantly different except for the cases of Soledad vs. Sincelejo (Mordecai method), Nevia vs. Sincelejo (Liu-Helmersson method) and Soledad vs. Sincelejo (Liu-Helmersson method).

## References

[1] Ferguson N.M. (2018). Challenges and opportunities in controlling mosquito-borne infections. Nature.

[2] Kraemer M.U., Sinka M.E., Duda K.A., Mylne A.Q., Shearer F.M., Barker C.M., Moore C.G., Carvalho R.G., Coelho G.E., Van Bortel W. (2015). The global distribution of the arbovirus vectors *Aedes aegypti* and *Ae. albopictus*. Elife.

[3] San Martín J.L., Brathwaite O., Zambrano B., Solórzano J.O., Bouckenooghe A., Dayan G.H., Guzmán M.G. (2010). The epidemiology of dengue in the americas over the last three decades: A worrisome reality. Am. J. Trop. Med. Hyg..

[4] Wartel T.A., Prayitno A., Hadinegoro S.R., Capeding M.R., Thisyakorn U., Tran N.H., Moureau A., Bouckenooghe A., Nealon J., Taurel A.F. (2017). Three Decades of Dengue Surveillance in Five Highly Endemic South East Asian Countries. Asia Pac. J. Public Health.

[5] Torres-Galicia I., Cortés-Poza D., Becker I. (2014). Dengue in Mexico: An analysis of two decades. Gac. Med. Mex..

[6] Barzon L. (2018). Ongoing and emerging arbovirus threats in Europe. J. Clin. Virol..

[7] Weaver S.C., Charlier C., Vasilakis N., Lecuit M. (2018). Zika, Chikungunya, and Other Emerging Vector-Borne Viral Diseases. Annu. Rev. Med..

[8] Yang H.M., Macoris M.L., Galvani K.C., Andrighetti M.T., Wanderley D.M. (2009). Assessing the effects of temperature on the population of *Aedes aegypti*, the vector of dengue. Epidemiol. Infect..

[9] Christophers S.R. (1960). Aedes aegypti (L.), the Yellow Fever Mosquito; Its Life History, Bionomics, and Structure.

[10] Mohammed A., Chadee D.D. (2011). Effects of different temperature regimens on the development of *Aedes aegypti* (L.) (Diptera: Culicidae) mosquitoes. Acta Trop..

[11] Reiskind M.H., Zarrabi A.A. (2012). Is bigger really bigger? Differential responses to temperature in measures of body size of the mosquito, Aedes albopictus. J. Insect. Physiol..

[12] Christofferson R.C., Mores C.N. (2016). Potential for Extrinsic Incubation Temperature to Alter Interplay Between Transmission Potential and Mortality of Dengue-Infected *Aedes aegypti*. Environ. Health Insights.

[13] Chan M., Johansson M.A. (2012). The incubation periods of Dengue viruses. PLoS ONE.

[14] Winokur O.C., Main B.J., Nicholson J., Barker C.M. (2020). Impact of temperature on the extrinsic incubation period of Zika virus in Aedes aegypti. PLoS Negl. Trop. Dis..

[15] Mbaika S., Lutomiah J., Chepkorir E., Mulwa F., Khayeka-Wandabwa C., Tigoi C., Oyoo-Okoth E., Mutisya J., Ng'ang'a Z., Sang R. (2016). Vector competence of Aedes aegypti in transmitting Chikungunya virus: Effects and implications of extrinsic incubation temperature on dissemination and infection rates. Virol. J..

[16] Westbrook C.J., Reiskind M.H., Pesko K.N., Greene K.E., Lounibos L.P. (2010). Larval environmental temperature and the susceptibility of Aedes albopictus Skuse (Diptera: Culicidae) to Chikungunya virus. Vector. Borne Zoonotic Dis..

[17] Christofferson R.C., Wearing H.J., Turner E.A., Walsh C.S., Salje H., Tran-Kiem C., Cauchemez S. (2022). How do i bite thee? let me count the ways: Exploring the implications of individual biting habits of *Aedes aegypti* for dengue transmission. PLoS Negl. Trop. Dis..

[18] Yasuno M., Tonn R.J. (1970). A study of biting habits of Aedes aegypti in Bangkok, Thailand. Bull. World Health Organ..

[19] Rueda L.M., Patel K.J., Axtell R.C., Stinner R.E. (1990). Temperature-dependent development and survival rates of *Culex quinquefasciatus* and *Aedes aegypti* (Diptera: Culicidae). J. Med. Entomol..

[20] Tun-Lin W., Burkot T.R., Kay B.H. (2000). Effects of temperature and larval diet on development rates and survival of the dengue vector *Aedes aegypti* in north Queensland, Australia. Med. Vet. Entomol..

[21] Tesla B., Demakovsky L.R., Mordecai E.A., Ryan S.J., Bonds M.H., Ngonghala C.N., Brindley M.A., Murdock C.C. (2018). Temperature drives *Zika virus* transmission: Evidence from empirical and mathematical models. Proc. Biol. Sci..

[22] Lusekelo E., Helikumi M., Kuznetsov D., Mushayabasa S. (2022). Modeling the effects of temperature and heterogeneous biting exposure on chikungunya virus disease dynamics. Inform. Med. Unlocked.

[23] Garrett-Jones C. (1964). Prognosis for interruption of malaria transmission through assessment of the mosquito's vectorial capacity. Nature.

[24] Smith D.L., Battle K.E., Hay S.I., Barker C.M., Scott T.W., McKenzie F.E. (2012). Ross, macdonald, and a theory for the dynamics and control of mosquito-transmitted pathogens. PLoS Pathog..

[25] Peña-García V.H., Triana-Chávez O., Arboleda-Sánchez S. (2017). Estimating Effects of Temperature on Dengue Transmission in Colombian Cities. Ann. Glob. Health.

[26] Pena-Garcia V.H., Christofferson R.C. (2019). Correlation of the basic reproduction number (R0) and eco-environmental variables in Colombian municipalities with chikungunya outbreaks during 2014–2016. PLoS Negl. Trop. Dis..

[27] Evans M.V., Hintz C.W., Jones L., Shiau J., Solano N., Drake J.M., Murdock C.C. (2019). Microclimate and Larval Habitat Density Predict Adult *Aedes albopictus* Abundance in Urban Areas. Am. J. Trop. Med. Hyg..

[28] Hayden M.H., Uejio C.K., Walker K., Ramberg F., Moreno R., Rosales C., Gameros M., Mearns L.O., Zielinski-Gutierrez E., Janes C.R. (2010). Microclimate and human factors in the divergent ecology of *Aedes aegypti* along the Arizona, U.S./Sonora, MX border. Ecohealth.

[29] Faridah L., Fauziah N., Agustian D., Mindra Jaya I.G.N., Eka Putra R., Ekawardhani S., Hidayath N., Damar Djati I., Carvajal T.M., Mayasari W. (2022). Temporal Correlation Between Urban Microclimate, Vector Mosquito Abundance, and Dengue Cases. J. Med. Entomol..

[30] Wimberly M.C., Davis J.K., Evans M.V., Hess A., Newberry P.M., Solano-Asamoah N., Murdock C.C. (2020). Land cover affects microclimate and temperature suitability for arbovirus transmission in an urban landscape. PLoS Negl. Trop. Dis..

[31] Murdock C.C., Evans M.V., McClanahan T.D., Miazgowicz K.L., Tesla B. (2017). Fine-scale variation in microclimate across an urban landscape shapes variation in mosquito population dynamics and the potential of *Aedes albopictus* to transmit arboviral disease. PLoS Negl. Trop. Dis..

[32] Scott T.W., Morrison A.C. (2010). Vector dynamics and transmission of dengue virus: Implications for dengue surveillance and prevention strategies: Vector dynamics and dengue prevention. Curr. Top. Microbiol. Immunol..

[33] López-Montenegro L.E., Pulecio-Montoya A.M., Marcillo-Hernández G.A. (2019). Dengue Cases in Colombia: Mathematical Forecasts for 2018–2022. MEDICC Rev..

[34] Gutierrez-Barbosa H., Medina-Moreno S., Zapata J.C., Chua J.V. (2020). Dengue Infections in Colombia: Epidemiological Trends of a Hyperendemic Country. Trop. Med. Infect. Dis..

[35] Liu-Helmersson J., Stenlund H., Wilder-Smith A., Rocklöv J. (2014). Vectorial capacity of *Aedes aegypti*: Effects of temperature and implications for global dengue epidemic potential. PLoS ONE.

[36] Mordecai E.A., Cohen J.M., Evans M.V., Gudapati P., Johnson L.R., Lippi C.A., Miazgowicz K., Murdock C.C., Rohr J.R., Ryan S.J. (2017). Detecting the impact of temperature on transmission of Zika, dengue, and chikungunya using mechanistic models. PLoS Negl. Trop. Dis..

[37] Ryan S.J., Carlson C.J., Mordecai E.A., Johnson L.R. (2019). Global expansion and redistribution of *Aedes*-borne virus transmission risk with climate change. PLoS Negl. Trop. Dis..

[38] Caminade C., Turner J., Metelmann S., Hesson J.C., Blagrove M.S., Solomon T., Morse A.P., Baylis M. (2017). Global risk model for vector-borne transmission of Zika virus reveals the role of El Niño 2015. Proc. Natl. Acad. Sci. USA.

[39] Turner J., Bowers R.G., Baylis M. (2013). Two-host, two-vector basic reproduction ratio (R(0)) for bluetongue. PLoS ONE.

[40] Vazquez-Prokopec G.M., Morrison A.C., Paz-Soldan V., Stoddard S.T., Koval W., Waller L.A., Alex Perkins T., Lloyd A.L., Astete H., Elder J. (2023). Inapparent infections shape the transmission heterogeneity of dengue. PNAS Nexus.

[41] Roy D., Borak J., Devadiga S., Wolfe R., Zheng M., Descloitres J. (2002). The MODIS land product quality assessment approach. Remote Sens. Environ..

[42] Landsat Quality Assessment ArcGIS Toolbox. https://www.usgs.gov/landsat-missions/landsat-quality-assessment-arcgis-toolbox.

[43] Avdan U., Jovanovska G. (2016). Algorithm for Automated Mapping of Land Surface Temperature Using LANDSAT 8 Satellite Data. J. Sens..

[44] Ciota A.T., Chin P.A., Ehrbar D.J., Micieli M.V., Fonseca D.M., Kramer L.D. (2018). Differential Effects of Temperature and Mosquito Genetics Determine Transmissibility of Arboviruses by *Aedes aegypti* in Argentina. Am. J. Trop. Med. Hyg..

[45] Bellone R., Failloux A.B. (2020). The Role of Temperature in Shaping Mosquito-Borne Viruses Transmission. Front. Microbiol..

[46] Samuel G.H., Adelman Z.N., Myles K.M. (2016). Temperature-dependent effects on the replication and transmission of arthropod-borne viruses in their insect hosts. Curr. Opin. Insect. Sci..

[47] Márquez Benítez Y., Monroy Cortés K.J., Martínez Montenegro E.G., Peña García V.H., Monroy Díaz Á.L. (2019). Influencia de la temperatura ambiental en el mosquito *Aedes* spp. y la transmisión del virus del dengue. Rev. CES Med..

[48] Peña-García V.H., Triana-Chávez O., Mejía-Jaramillo A.M., Díaz F.J., Gómez-Palacio A., Arboleda-Sánchez S. (2016). Infection Rates by Dengue Virus in Mosquitoes and the Influence of Temperature May Be Related to Different Endemicity Patterns in Three Colombian Cities. Int. J. Environ. Res. Public Health.

[49] Felix G.E., Barrera R., Vazquez J., Ryff K.R., Munoz-Jordan J.L., Matias K.Y., Hemme R.R. (2018). Entomological Investigation of *Aedes aegypti* in Neighborhoods with Confirmed Human Arbovirus Infection in Puerto Rico. J. Am. Mosq. Control Assoc..

[50] Dos Santos T.P., Cruz O.G., da Silva K.A.B., de Castro M.G., de Brito A.F., Maspero R.C., de Alcântra R., Dos Santos F.B., Honorio N.A., Lourenço-de-Oliveira R. (2017). Dengue serotype circulation in natural populations of *Aedes aegypti*. Acta Trop..

[51] Pérez-Castro R., Castellanos J.E., Olano V.A., Matiz M.I., Jaramillo J.F., Vargas S.L., Sarmiento D.M., Stenström T.A., Overgaard H.J. (2016). Detection of all four dengue serotypes in *Aedes aegypti* female mosquitoes collected in a rural area in Colombia. Mem. Inst. Oswaldo Cruz.

[52] Kirstein O.D., Ayora-Talavera G., Koyoc-Cardeña E., Chan Espinoza D., Che-Mendoza A., Cohuo-Rodriguez A., Granja-Pérez P., Puerta-Guardo H., Pavia-Ruz N., Dunbar M.W. (2021). Natural arbovirus infection rate and detectability of indoor female *Aedes aegypti* from Mérida, Yucatán, Mexico. PLoS Negl. Trop. Dis..

[53] Anders K.L., Nga le H., Thuy N.T., Ngoc T.V., Tam C.T., Tai L.T., Truong N.T., Duyen H.T., Trung V.T., Kien D.T. (2015). Households as foci for dengue transmission in highly urban Vietnam. PLoS Negl. Trop. Dis..

[54] Stoddard S.T., Forshey B.M., Morrison A.C., Paz-Soldan V.A., Vazquez-Prokopec G.M., Astete H., Reiner R.C., Vilcarromero S., Elder J.P., Halsey E.S. (2013). House-to-house human movement drives dengue virus transmission. Proc. Natl. Acad. Sci. USA.

[55] Liebman K.A., Stoddard S.T., Morrison A.C., Rocha C., Minnick S., Sihuincha M., Russell K.L., Olson J.G., Blair P.J., Watts D.M. (2012). Spatial dimensions of dengue virus transmission across interepidemic and epidemic periods in Iquitos, Peru (1999–2003). PLoS Negl. Trop. Dis..

[56] Brown J., Pascual M., Wimberly M., Johnson L., Murdock C. (2023). Humidity-The Overlooked Variable in Thermal Biology of Mosquito-Borne Disease. Authorea.

[57] Carrington L.B., Seifert S.N., Armijos M.V., Lambrechts L., Scott T.W. (2013). Reduction of Aedes aegypti vector competence for dengue virus under large temperature fluctuations. Am. J. Trop. Med. Hyg..

[58] Lambrechts L., Paaijmans K.P., Fansiri T., Carrington L.B., Kramer L.D., Thomas M.B., Scott T.W. (2011). Impact of daily temperature fluctuations on dengue virus transmission by Aedes aegypti. Proc. Natl. Acad. Sci. USA.

[59] Carrington L.B., Armijos M.V., Lambrechts L., Scott T.W. (2013). Fluctuations at a low mean temperature accelerate dengue virus transmission by Aedes aegypti. PLoS Negl. Trop. Dis..

[60] Power G.M., Vaughan A.M., Qiao L., Sanchez Clemente N., Pescarini J.M., Paixao E.S., Lobkowicz L., Raja A.I., Portela Souza A., Barreto M.L. (2022). Socioeconomic risk markers of arthropod-borne virus (arbovirus) infections: A systematic literature review and meta-analysis. BMJ Glob. Health.

[61] Carrasquilla M.C., Ortiz M.I., Leon C., Rondon S., Kulkarni M.A., Talbot B., Sander B., Vasquez H., Cordovez J.M., Gonzalez C. (2021). Entomological characterization of Aedes mosquitoes and arbovirus detection in Ibague, a Colombian city with co-circulation of Zika, dengue and chikungunya viruses. Parasit. Vectors.

[62] Carabali M., Lim J.K., Palencia D.C., Lozano-Parra A., Gelvez R.M., Lee K.S., Florez J.P., Herrera V.M., Kaufman J.S., Rojas E.M. (2018). Burden of dengue among febrile patients at the time of chikungunya introduction in Piedecuesta, Colombia. Trop. Med. Int. Health.

[63] Carabali M., Maheu-Giroux M., Kaufman J.S. (2021). Dengue, Severity Paradox, and Socioeconomic Distribution Among Afro-Colombians. Epidemiology.

[64] Toan N.T., Rossi S., Prisco G., Nante N., Viviani S. (2015). Dengue epidemiology in selected endemic countries: Factors influencing expansion factors as estimates of underreporting. Trop. Med. Int. Health.

[65] Sanabria-Mazo J.P., Useche-Aldana B., Ochoa P.P., Rojas-Gualdron D.F., Mateo-Canedo C., Carmona-Cervello M., Crespo-Puig N., Selva-Olid C., Muro A., Mendez-Ulrich J.L. (2021). Social Inequities in the Impact of COVID-19 Lockdown Measures on the Mental Health of a Large Sample of the Colombian Population (PSY-COVID Study). J. Clin. Med..

[66] De la Rosa A., Monterrosa Quintero A., Camacho-Villa M.A., Arc-Chagnaud C., Andrade A.G.P., Reyes-Correa S., Quintero-Bernal R., Fuentes-Garcia J.P. (2022). Physical Activity Levels and Psychological Well-Being during COVID-19 Lockdown among University Students and Employees. Int. J. Environ. Res. Public Health.

[67] Wilder-Smith A. (2021). Dengue during the COVID-19 pandemic. J. Travel. Med..

[68] Sasmono R.T., Santoso M.S. (2022). Movement dynamics: Reduced dengue cases during the COVID-19 pandemic. Lancet Infect. Dis..

[69] Acosta-Pérez T., Rodríguez-Yánez T., Almanza-Hurtado A., Martínez-Ávila M.C., Dueñas-Castell C. (2022). Dynamics of dengue and SARS-CoV-2 co-infection in an endemic area of Colombia. Trop. Dis. Travel. Med. Vaccines.

[70] Khan S., Akbar S.M.F., Yahiro T., Mahtab M.A., Kimitsuki K., Hashimoto T., Nishizono A. (2022). Dengue Infections during COVID-19 Period: Reflection of Reality or Elusive Data Due to Effect of Pandemic. Int. J. Environ. Res. Public Health.

