# Supplementary material for: Arbovirus Transmission Predictions Are Affected by Both Temperature Data Source and Modeling Methodologies across Cities in Colombia
Source: Microorganisms. 2023 May 9;11(5):1249. doi: 10.3390/microorganisms11051249 (PMC10223750; doi:10.3390/microorganisms11051249)

Supplemental File S1

Micro-environmental temperatures and R0, Peña-Garcia et al.

Figure S1.1: Values recorded by temperature data loggers in the three cities at six different hours along the day.

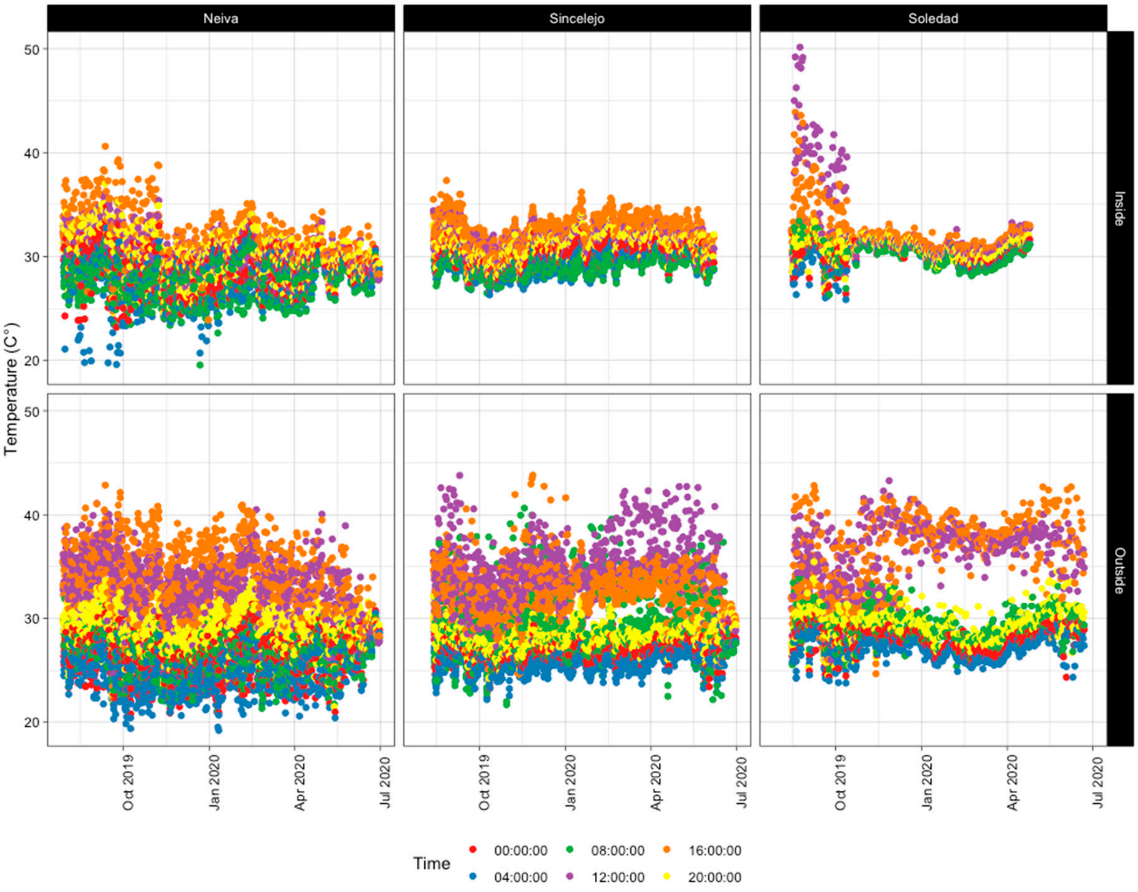

Supplemental Table S1.1: Results of t-test comparison analyses between daily mean temperature values taken indoor (IHT), outdoor (OHT) and weather stations (WST). *p*-values in bold italic are statistically significant at 95% confidence.

City	House	Comparison	t	*p*-value
Neiva	NE1	IHT vs OHT	1.670	0.095
		IHT vs WST	-5.165	***3.26E-07***
		OHT vs WST	-6.946	***8.61E-12***
	NE2	IHT vs OHT	1.655	0.101
		IHT vs WST	5.017	***1.02E-05***
		OHT vs WST	2.187	***0.031***
	NE3	IHT vs OHT	3.010	***0.003***
		IHT vs WST	9.734	***4.41E-21***
		OHT vs WST	7.053	***4.25E-12***
	NE4	OHT vs WST	0.277	0.782
	NE5	IHT vs OHT	11.472	***4.89E-24***
		IHT vs WST	16.381	***3.45E-38***
		OHT vs WST	5.431	***8.32E-08***
Sincelejo	SI1	IHT vs OHT	5.386	***1.59E-07***
		IHT vs WST	19.486	***1.32E-57***
		OHT vs WST	10.384	***2.74E-21***
	SI2	IHT vs OHT	12.541	***7.46E-31***
		IHT vs WST	16.835	***2.79E-50***
		OHT vs WST	5.606	***3.01E-08***
	SI3	OHT vs WST	9.759	***4.04E-21***
	SI4	IHT vs OHT	14.356	***2.15E-40***
		IHT vs WST	28.709	***5.16E-118***
		OHT vs WST	12.370	***8.27E-32***
	SI5	IHT vs OHT	12.267	***8.49E-27***
		IHT vs WST	24.035	***3.23E-92***
		OHT vs WST	4.201	***3.74E-05***
Soledad	SO2	IHT vs OHT	13.049	***5.21E-27***
	SO3	IHT vs OHT	3.821	***0.002***

Supporting Table S1.2: Summary of linear associations between OHT and IHT daily-averaged temperatures. (Model: IHT = OHT)

City	Month	Intercept	Slope	R^2
Neiva	jul-19	10.9391837	0.64602022	0.80
	aug-19	6.75190908	0.78937529	0.71
	sep-19	7.08511394	0.78605929	0.79
	oct-19	9.14904805	0.70472738	0.83
	nov-19	3.06841794	0.90694088	0.90
	dec-19	19.0461326	0.31485739	0.24
	jan-20	11.8745302	0.59715459	0.59
	feb-20	8.10444521	0.73249626	0.93
	mar-20	11.357985	0.61376135	0.85
	apr-20	1.23906931	0.97915962	0.69
	may-20	12.7036514	0.58785226	0.64
	jun-20	14.0789538	0.52246391	0.69
Sincelejo	aug-19	10.7956842	0.68067997	0.93
	sep-19	7.52881355	0.78505737	0.83
	oct-19	8.61310222	0.73656881	0.83
	nov-19	10.7403438	0.66392409	0.81
	dec-19	16.4451649	0.48178219	0.61
	jan-20	9.70132749	0.71903774	0.78
	feb-20	10.4578667	0.70018186	0.83
	mar-20	14.8638782	0.55712997	0.59
	apr-20	11.6499929	0.65007331	0.83
	may-20	13.3334245	0.58757893	0.77
	jun-20	17.5570976	0.42604734	0.73
Soledad	aug-19	5.79725172	0.88204092	0.81
	sep-19	6.85997423	0.83434497	0.95
	oct-19	13.6651609	0.59936162	0.67
	nov-19	23.2756706	0.26899443	0.46
	dec-19	25.4461461	0.18043655	0.24
	jan-20	15.60591	0.46743913	0.53
	feb-20	13.2720983	0.53794684	0.56
	mar-20	14.1821195	0.51395315	0.24
	apr-20	20.5418323	0.34254735	0.16

Supporting Fig. S1.2: Regression analyses performed on HOBO-recorded outdoor and indoor daily averages for each month and each city.

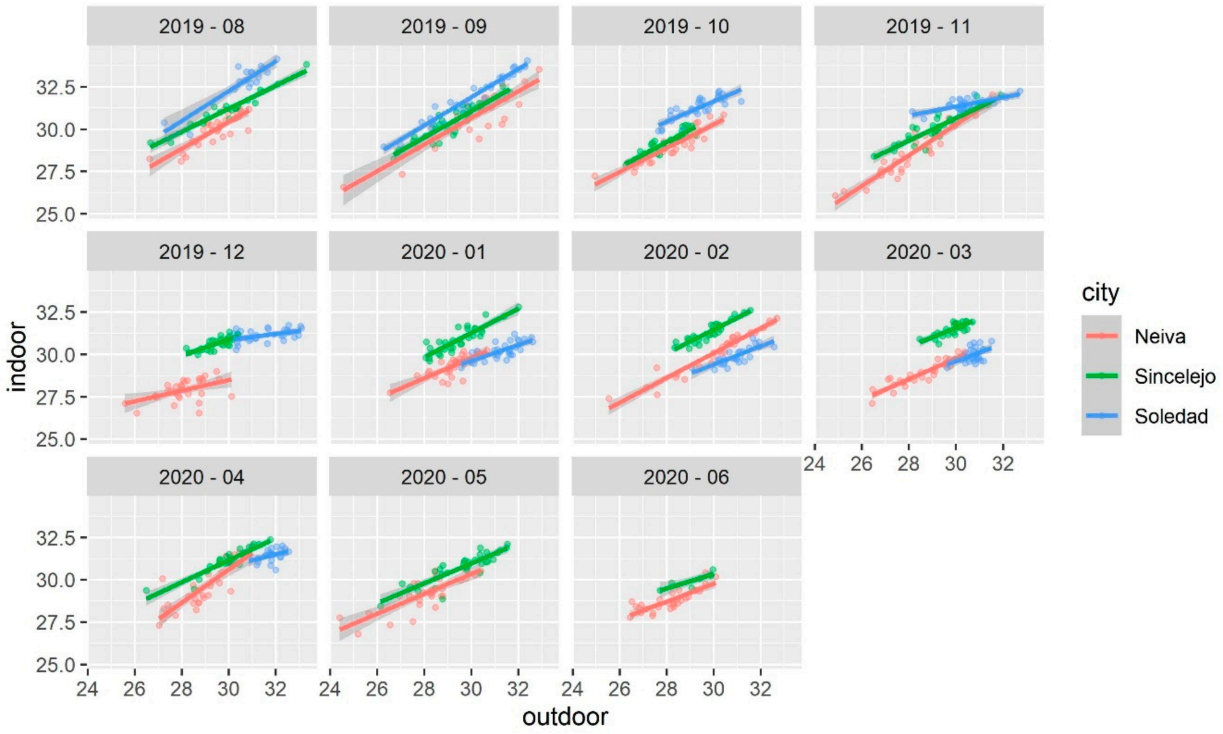

Supplement: Supplementary file 1 [file microorganisms-11-01249-s001.zip › SuppFileS1.pdf]
