# Supplementary material for: Arbovirus Transmission Predictions Are Affected by Both Temperature Data Source and Modeling Methodologies across Cities in Colombia
Source: Microorganisms. 2023 May 9;11(5):1249. doi: 10.3390/microorganisms11051249 (PMC10223750; doi:10.3390/microorganisms11051249)

**Supplemental File S2: Temperature Dependence in Model Parameters**

*Micro-environmental temperatures and R0*, Peña-Garcia et al.

Mosquito density was derived by Caminade from data in Kraemer et al 2015. Those values are as follows: For *Ae. aegypti* in the city of Neiva, mosquito density (m1) was 731.721069, while in Sincelejo, m1=752.021729, and in Soledad, m1 = 894.557617. For *Ae. albopictus*, m2 = 668.480164 for Neiva, m2= 383.251526 for Sincelejo, and m2= 177.526230 for Soledad. We used these same estimates to initialize adult mosquito density for derivation of temperature dependent adult mosquito density in the R0 calculations for the Caminade and the Liu-Helmersson methods.

Across the three methods there were commonalities in included parameters:

- Biting rate
    - a for Liu-Helmersson and Mordecai methods
    - a1 for method from Caminade
- Vector competence
    - b from Liu-Helmersson
    - bm from Caminade (transmission probability – host to vector)
    - c from Mordecai (probability mosquito becomes infected given exposure)
- Extrinsic Incubation Period (EIP)
    - EIP for Liu-Helmersson and Caminade
    - 1/EIR for Mordecai where EIR = extrinsic incubation rate
- Mosquito mortality
    - mu for Caminade and Liu-Helmersson
    - 1/lf for Mordecai where lf = lifespan in days of the mosquito

Below are the comparisons of the shared parameters across methods with respect to temperature sources and cities.

**Figure S2.1:** Temperature-dependent estimate of biting rate of *Ae. aegypti*

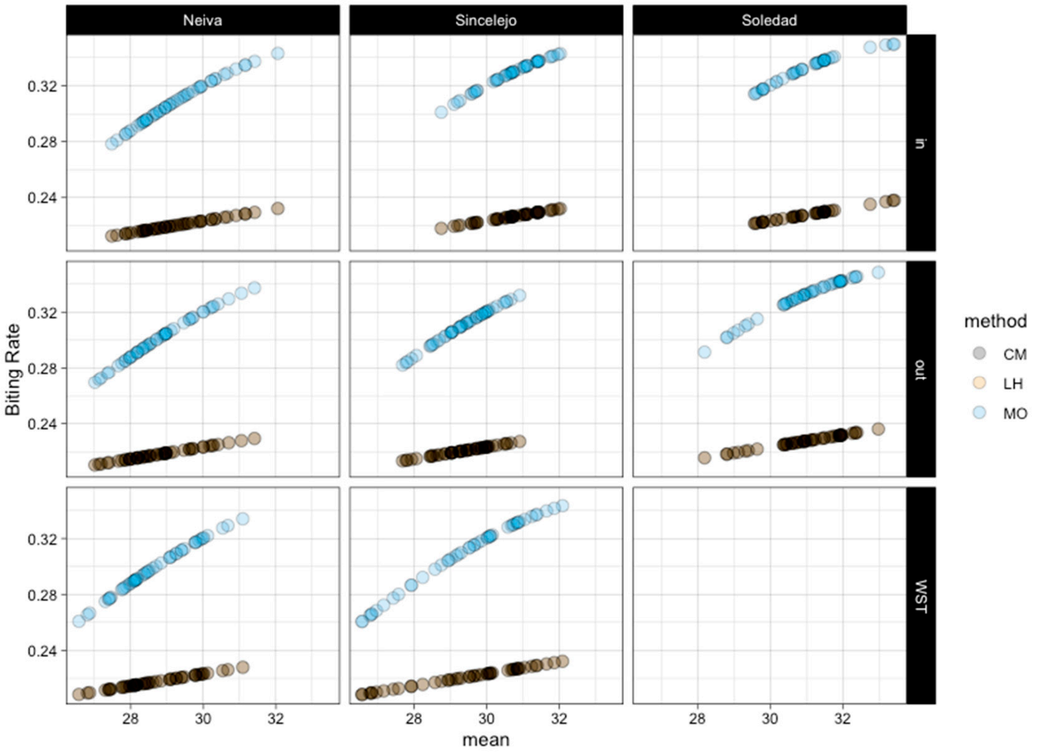

**Figure S2.2:** Temperature-dependent estimate of the probability of successful transmission from human to mosquito (of *Ae. aegypti)* as an estimate for vector competence.

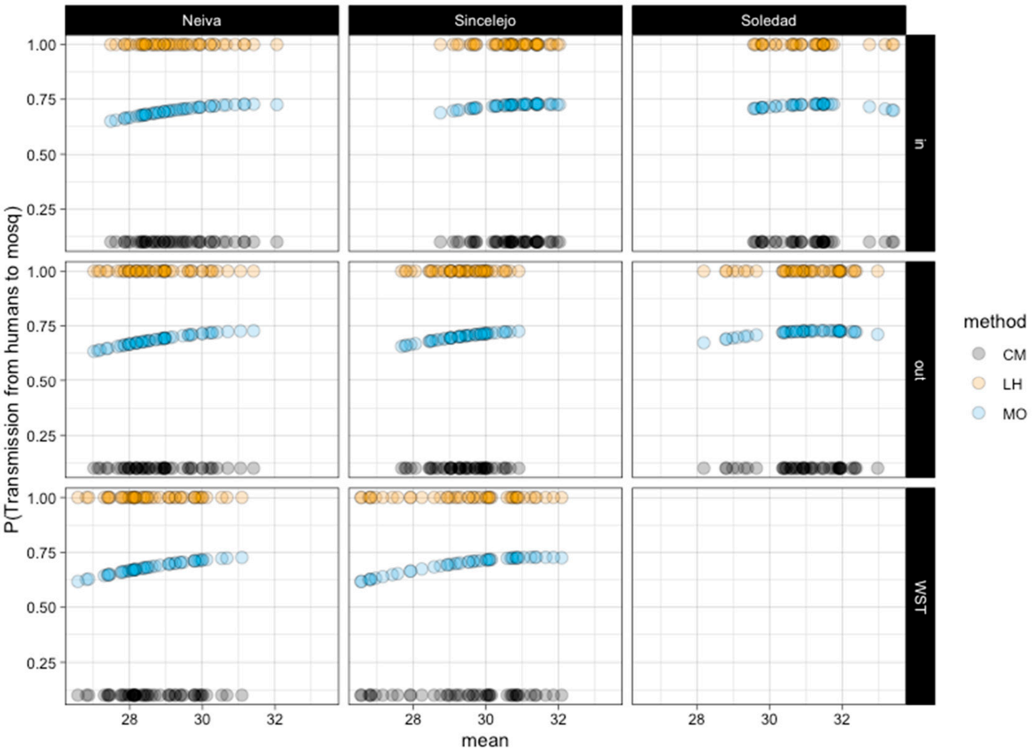

**Figure S2.3:** Temperature-dependent estimate of the extrinsic incubation period of *Ae. aegypti*

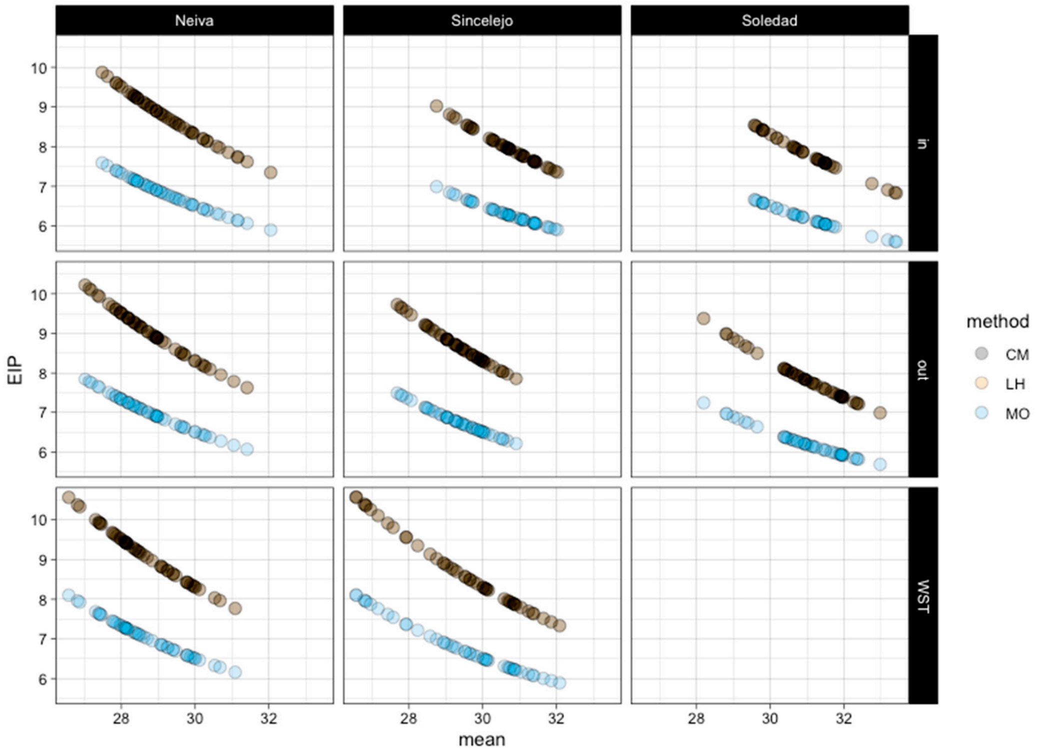

**Figure S2.4:** Temperature-dependent estimate of the mosquito mortality of *Ae. aegypti*

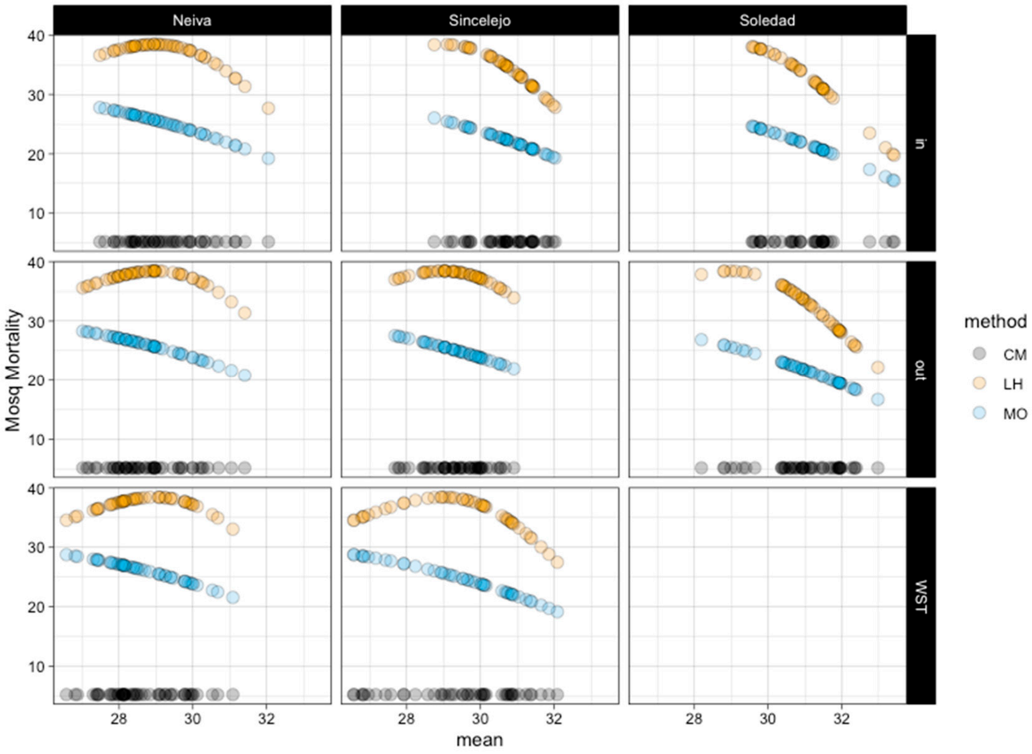

**Figure S2.5: Temperature-dependent estimate of the R0 for each method**

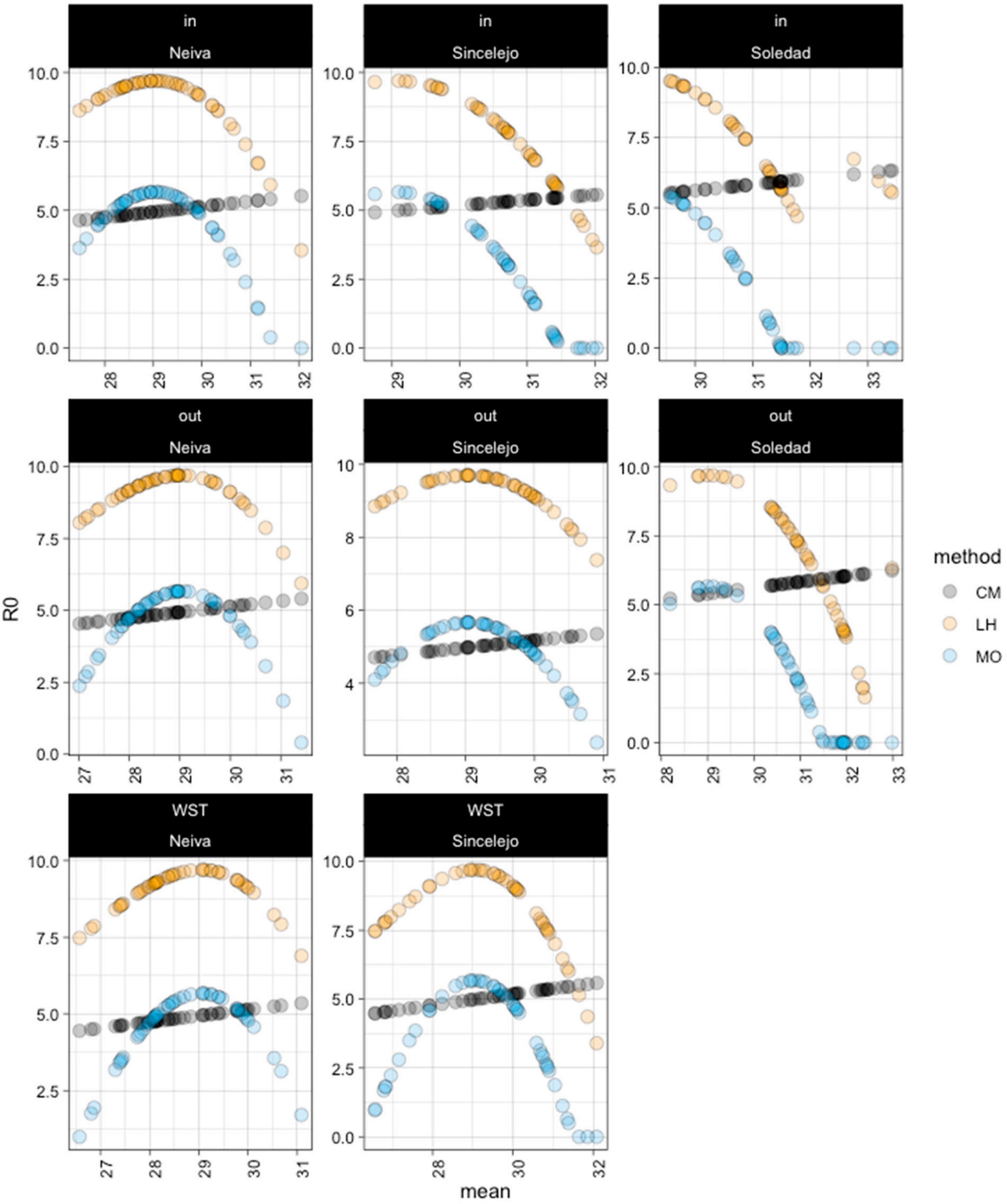

**Figure S2.6:** Distributions of the R0 values per city x data source x modeling method. All distributions were significantly different except for the cases of Soledad vs. Sincelejo (Mordecai method), Nevia vs. Sincelejo (Liu-Helmersson method) and Soledad vs. Sincelejo (Liu-Helmersson method).

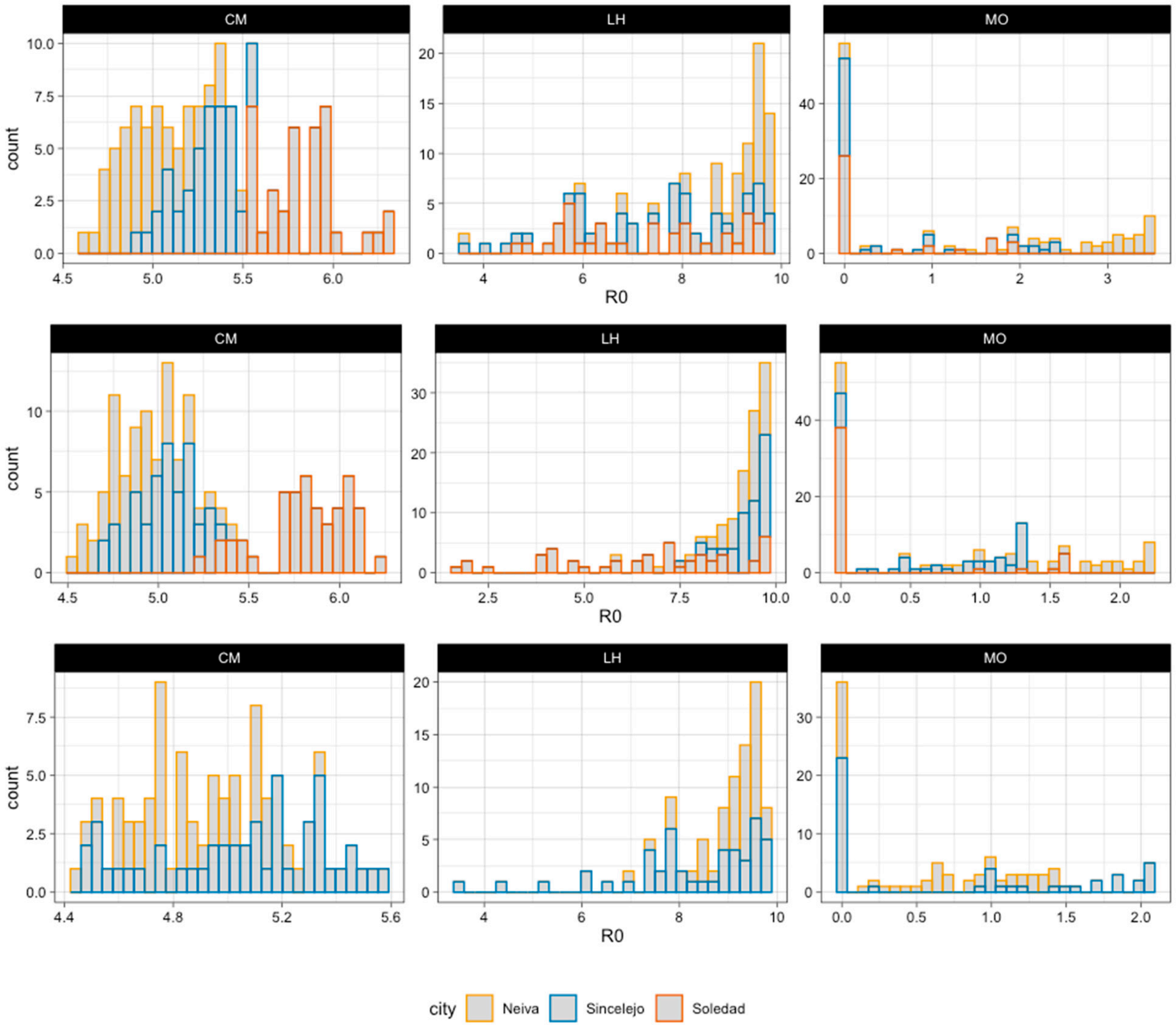

Supplement: Supplementary file 1 [file microorganisms-11-01249-s001.zip › SuppFileS2.pdf]
